# Characteristics and Outcomes of Intracranial Hemorrhage in Cancer Patients Visiting the Emergency Department

**DOI:** 10.3390/jcm11030643

**Published:** 2022-01-27

**Authors:** Aiham Qdaisat, Sai-Ching J. Yeung, Cristhiam H. Rojas Hernandez, Pavani Samudrala, Mona Kamal, Ziyi Li, Adriana H. Wechsler

**Affiliations:** 1 Department of Emergency Medicine, The University of Texas MD Anderson Cancer Center, Houston, TX 77030, USA; aqdaisat@mdanderson.org (A.Q.); syeung@mdanderson.org (S.-C.J.Y.); pavanigmc@gmail.com (P.S.); 2 Section of Benign Hematology, The University of Texas MD Anderson Cancer Center, Houston, TX 77030, USA; cmrojas@mdanderson.org; 3 Symptom Research Department, The University of Texas MD Anderson Cancer Center, Houston, TX 77030, USA; mkjomaa@mdanderson.org; 4 Department of Biostatistics, The University of Texas MD Anderson Cancer Center, Houston, TX 77030, USA; zli16@mdanderson.org

**Keywords:** cancer, intracranial hemorrhages, emergency, mortality, platelet count, characteristics, outcome

## Abstract

Intracranial hemorrhage (ICH) is a dreaded complication of both cancer and its treatment. To evaluate the characteristics and clinical outcomes of cancer patients with ICH, we identified all patients with ICH who visited The University of Texas MD Anderson Cancer Center emergency department between 1 September 2006 and 16 February 2016. Clinical and radiologic data were collected and compared. Logistic regression analyses were used to determine the association between clinical variables and various outcomes. During the period studied, 704 confirmed acute ICH cases were identified. In-hospital, 7-day, and 30-day mortality rates were 15.1, 11.4, and 25.6%, respectively. Hypertension was most predictive of intensive care unit admission (OR = 1.52, 95% CI = 1.09–2.12, *p* = 0.013). Low platelet count was associated with both in-hospital mortality (OR = 0.96, 95% CI = 0.94–0.99, *p* = 0.008) and 30-day mortality (OR = 0.98, 95% CI = 0.96–1.00, *p* = 0.016). Radiologic findings, especially herniation and hydrocephalus, were strong predictors of short-term mortality. Among known risk factors of ICH, those most helpful in predicting cancer patient outcomes were hypertension, low platelet count, and the presence of hydrocephalus or herniation. Understanding how the clinical presentation, risk factors, and imaging findings correlate with patient morbidity and mortality is helpful in guiding the diagnostic evaluation and aggressiveness of care for ICH in cancer patients.

## 1. Introduction

Intracranial hemorrhage (ICH) is a dreaded complication of both cancer and its treatment. In the general population, intracerebral hemorrhage represents only 10–20% of strokes but is more deadly, with a reported case fatality ratio of 24–37% at 7 days and 40–59% at 30 days [1,2,3,4,5,6]. Among cancer patients, both the incidence of ICH and ICH-associated mortality rates are assumed to be increased, heightening the need for a clear understanding of the characteristics of cancer patients with ICH and how these characteristics affect outcomes.

Studies to date have been limited in terms of either inclusion criteria or scope of variables investigated, such that the relative contribution of specific variables to outcomes is not discernable. Examining a large database of hospitalized patients, Murthy et al. concluded that cancer patients with ICH had higher in-hospital mortality rates and higher morbidity rates than non-cancer patients. However, patients with cerebral metastases or primary brain tumors were excluded from that analysis. Some studies considered only liquid tumors or primary CNS tumors, thus increasing susceptibility to bias effects [7,8,9,10]. Other studies focus on one risk factor, such as anticoagulation therapy, without consideration for its interplay with others [9,11].

In the current study, we take a more comprehensive look at the characteristics, management, and clinical outcomes of ICH in cancer patients presenting to the emergency department (ED) of a dedicated cancer center. Our large cancer population allowed us to discriminate outcomes by cancer type, presence of metastases, and presence of risk factors. Understanding which patient characteristics and risk factors carry the best prognosis can help ED physicians identify the patients most vulnerable to ICH and expedite appropriate evaluation and management in those first critical hours of hematoma expansion. Alternatively, identifying the patients least likely to survive can limit aggressive interventions that may be futile and instead turn attention to goals of care.

## 2. Materials and Methods

### 2.1. Study Participants and Data Collection

Under an approved institutional review board protocol, we performed a retrospective cohort study of all consecutive patients who visited the ED of The University of Texas MD Anderson Cancer Center in Houston, TX, USA, between 1 September 2006 and 16 February 2016, with a diagnosis of ICH. Eligible cases were identified from the institutional billing database using all ICH related ICD-9 and ICD-10 codes. Exclusion criteria were (1) age younger than 18 years, (2) no cancer diagnosis, (3) no acute ICH present, (4) ICH diagnosed at another institution, (5) patients transferred out, and (6) missing ED physician notes.

Cases were reviewed by four independent investigators using the electronic medical record system and a defined data dictionary. All investigators were trained on data extraction and used a standardized form to guide data collection. Finally, fifty charts were randomly selected and reviewed by a different abstractor than the original abstractor to assess the interrater agreement, reporting the Cohen’s kappa coefficient (κ). Patient demographics, clinical and laboratory findings, cancer-related factors, and comorbidities that are known to be associated with ICH were collected through chart review. Imaging reports were reviewed for confirmation of acute ICH, defined as a subdural, epidural, intraparenchymal, or subarachnoid bleed, as well as a hemorrhagic brain tumor, found on diagnostic brain CT or MRI within 24 h of the ED visit. The first available value for the laboratory studies was used for the analysis as continuous variables.

### 2.2. Statistical Analysis

Descriptive statistics were used to describe and compare the characteristics of the cohort. Significance was appraised using the chi-square test, Fisher’s exact test, Student’s *t*-test, or non-parametric tests (Wilcoxon–Mann–Whitney test) when normality assumption was not met. Univariate logistic regression analysis was used to determine the association between each of the clinical and cancer-related variables and ICH outcomes. Significant variables from the univariate analyses were further analyzed using a multiple logistic regression model. Variables with more than 20% missing values were not included in the analyses. A two-tailed *p*-value < 0.05 was considered statistically significant. All statistical analyses were performed using R software (version 3.6.3, The R Foundation, Available online: http://www.r-project.org (accessed on 11 March 2020)).

### 2.3. Study Cohort and Interobserver Agreement

**Figure 1:**
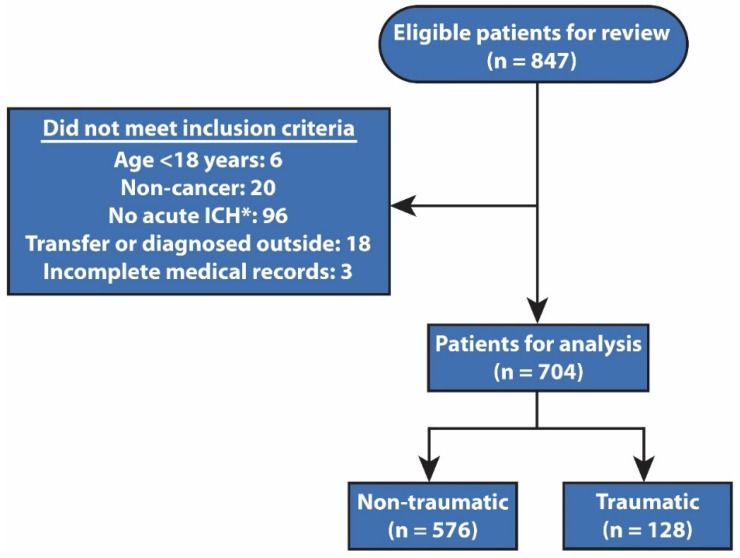
Exclusion criteria used to determine study eligibility. Abbreviations: ICH, intracranial hemorrhage. * Patients with intracranial hemorrhage that was chronic, resolved, or patients with postoperative changes were also excluded.

During the 10-year period studied, 77,925 unique cancer patients made 204,464 ED visits to a large, urban National Cancer Institute–designated comprehensive cancer center, serving mostly oncology patients. A total of 704 patients had confirmed acute ICH once the eligibility criteria were applied (0.34% of ED visits). The kappa coefficient (κ) for the interrater agreement was 0.873, indicating a very-good agreement between different abstractors. Among the 704 patients with confirmed acute ICH, 128 (18.2%) had traumatic ICH and the remaining 576 (81.8%) had spontaneous ICH (Figure 1).

## 3. Results

### 3.1. Characteristics of Cancer Patients with ICH Presenting to an Oncologic ED

The most common underlying cancer types were leukemia (27.8%) and melanoma (17.5%). Most of the patients (88.8%) had active cancer. Patients with traumatic ICH were significantly older than those with spontaneous ICH (median age 66 years compared with 59 years; *p* < 0.001). Furthermore, history of hypertension (53.1% compared with 42.4%; *p* = 0.034), hypercholesterolemia (28.1% compared with 17.4%; *p* = 0.008), and hematologic malignancies (54.7% compared with 31.3%; *p* < 0.001) were observed significantly more frequently among patients with traumatic ICH than among those with spontaneous ICH (Table 1). Headache (42.2%) and altered mental status (35.9%) were the most common presenting symptoms (Table S1).

Subdural hematoma was the most common (35.2%) location for ICH. Other frequent locations were hemorrhagic metastasis (28.3%), intraparenchymal hemorrhage (10.5%), and subarachnoid hemorrhage (8.8%). Common associated imaging findings were edema (47.2%) and midline shift (34.2%). Ninety-three patients (13.2%) had herniation and 77 patients (10.9%) had hydrocephalus (Table S2). Subfalcine herniation was the most common type of herniation (5.0%) identified, followed by uncal herniation (4.3%; Table S3). Cerebellar tonsillar herniation was identified in 2% of the patients. Melanoma (47.2%) and lung cancer (18.6%) were the most common primary tumors associated with intracranial metastatic hemorrhagic lesions (Table S4). Intra-metastatic bleeding was associated with a lower incidence of midline shift and herniation (Table S5).

Most patients were admitted to either a hospital ward (50.6%) or an intensive care unit (ICU; 41.5%). Thirty-five patients (5%) were discharged to home or to hospice, only 6 (0.9%) were directly transferred to the operating room for surgery, and 4 (0.6%) died in the ED. Treatment was initiated in most patients (93.8%), and platelet transfusion was the most common treatment modality (30%), followed by dexamethasone (20.3%), whereas early surgical intervention was uncommon (11.7%, Table 2).

### 3.2. Risk Factors for ICU Admission and Prolonged Hospital Stay in Cancer Patients with ICH

The median hospital length of stay for the whole population was 5 days, and it was significantly higher in patients with traumatic ICH than in those with spontaneous ICH (6 days compared with 5 days; *p* = 0.007). Among the 292 patients (41.5%) who were admitted to the ICU (Table 2), 230 had spontaneous ICH, representing 39.9% of the spontaneous ICH group (230/576), and 62 had traumatic ICH, representing 48.4% of that group (62/128). The median ICU length of stay was 2 days for both subgroups (Table 2).

History of hypertension and low platelet count were associated with longer hospital stay (hypertension: odds ratio (OR) = 4.77, 95% confidence interval (CI) = 1.30–22.70, *p* = 0.045; low platelets: OR = 0.93, 95% CI = 0.87–1.00, *p* = 0.041; Table 3 and Table S6). Hypertension was also associated with an increased likelihood of being admitted to the ICU (OR = 1.52, 95% CI = 1.09–2.12, *p* = 0.013), and intratumor bleeding was associated with a decreased likelihood of being admitted to the ICU (OR = 0.66, 95% CI = 0.45–0.97, *p* = 0.033). Patients with a platelet count less than 50 K/uL had significantly longer hospital stay and ICU admission (OR = 27.96, 95% CI = 5.20–150.4, *p* < 0.001 and OR = 2.22, 95% CI = 1.60–3.09, *p* < 0.001: respectively).

### 3.3. Mortality in Cancer Patients with ICH

**Figure 2:**
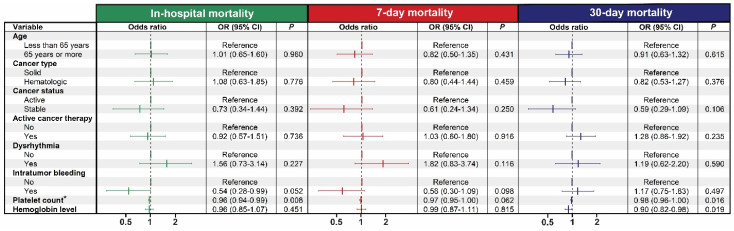
Multivariable analysis of the association between clinical factors and short-term mortality in cancer patients with intracranial hemorrhage. Platelet count was calculated as a continuous variable in increments of 10 K/uL. Abbreviations: OR, odds ratio; CI, confidence interval.

One hundred six patients (15.1%) died during their hospital stay. Seven-day and 30-day mortality rates were 11.4% and 25.6%, respectively (Table 2). Multiple myeloma, sarcoma, gastrointestinal tumors, and leukemia had the highest 7-day and 30-day mortality (Table S7). Mortality rates and ICU admissions were significantly different according to the type of intracranial hemorrhage with the highest mortality rates observed in patients with intraparenchymal hemorrhage (Table S8). In the univariate analysis, several clinical factors were associated with short-term mortality (Table S9). Significant variables from the univariate analysis were further investigated using a multivariable logistic regression model (Figure 2). Platelet count and hemoglobin level were significant factors that predicted short-term mortality. Low platelet count was associated with increased in-hospital mortality rate (OR = 0.96, 95% CI = 0.94–0.99, *p* = 0.008) and 30-day mortality rate (OR = 0.98, 95% CI = 0.96–1.00, *p* = 0.016), indicating that a drop in platelet count by a magnitude of 10 K/uL increased the risk of mortality by 2–4%. Similar results were observed when the platelet count was categorized using a cutoff point of 50 K/uL (Table S9). In the same manner, low hemoglobin level was associated with increased 30-day mortality rate (OR = 0.90, 95% CI = 0.82–0.98, *p* = 0.019), but not with in-hospital or 7-day mortality rates.

To determine the effect of various radiologic findings on short-term mortality, we constructed additional logistic regression models (Table S10 and Table 4). As each radiologic finding correlated with the others (*p* < 0.001 for all), and to prevent multicollinearity in the model, we constructed logistic regression models for each radiologic finding separately, controlling for platelet count, hemoglobin level, and intratumor bleeding (Table 4). Midline shift, edema, herniation, and hydrocephalus were all significantly associated with an increased risk of short-term mortality after adjusting for platelet count, hemoglobin level, and intratumor bleeding. The presence of herniation or hydrocephalus was associated with increased risk of 7 days mortality (adjusted odds ratio [AOR] = 10.63, 95% CI = 6.18–18.48, *p* < 0.001) and (AOR = 5.78, 95% CI = 3.19–10.39, *p* < 0.001) respectively (Table 4).

## 4. Discussion

To the best of our knowledge, our population is the largest cohort of cancer patients with ICH to be studied to date, with more than 700 patients, compared with 208 in a 2010 study from another cancer center [12]. ICH remains an uncommon diagnosis for cancer patients despite their increased risk. Only 0.34% of all ED visits over the 10-year period studied involved ICH. Our cancer population also had a significantly lower inpatient mortality rate (15.1%) than previously reported (37.3%) in a large hospital database review of cancer patients from 2016, roughly the same period as our study [7]. The higher mortality rate in the hospital study may be a result of the exclusion of patients with brain tumors since we found that patients with intratumor bleeding often had a shorter hospital stay and lower rates of ICU admission, in-hospital mortality, and 7-day mortality. Hemorrhage into metastases was the most common type of bleeding in patients with spontaneous ICH. Intratumor bleeding also appeared to be more benign in terms of 30-day mortality rates according to our multivariable analysis, as noted in previous studies [12]. This has also been found to be true of primary brain tumors. Although some primary brain tumors such as glioblastomas are extremely thrombogenic and also pathophysiologically prone to bleeding, their increased risk of hemorrhage did not affect survival. In fact, overall survival was higher for patients receiving anticoagulation for VTE [13]. Furthermore, Burth et al. showed that the use of anticoagulants did not alter the risk of intracranial hemorrhage among patients with brain tumors (both primary and metastatic) [14]. We hypothesize that intra-metastatic bleeding prognosticates a more benign course because they are often smaller and better accommodated in the fixed cranial space. Although we do not have volumetric measurements of the bleeding due to variability of reporting in this retrospective review, we used the presence of herniation or midline shift as a surrogate marker for larger, space-occupying bleeds.

Patients with traumatic ICH tended to be older, and proportionally more of them had hematologic malignancies. The most common location by far was subdural hematoma (61.7%) since subdural bleeds tend to be provoked by trauma, and the thrombocytopenia accompanying leukemia places these patients at greater risk. Traumatic ICH also resulted in slightly higher ICU admission rates and significantly longer hospital stays, reflecting more severe or complication-laden bleeds.

The in-hospital mortality rate was 15.1% or our patients, close to the 22% noted in the Navi et al. study from 2010, which, like our cohort, included patients with brain metastases and primary brain tumors [12]. As in our study, Murthy et al. reported that the 7-day mortality rate from ICH was almost twice as high in patients with hematologic malignancies as in those with solid or central nervous system tumors, but by 30 days, that difference was narrowing, and by 1 year, the mortality rate was nearly equivalent between patients with hematologic and solid tumors [7]. This may reflect the fact that by 30 days or 1 year, one can no longer confidently attribute mortality to the hemorrhagic event; death may instead be a result of complications of the malignancy itself.

Morbidity was also higher in patients with hematologic malignancies. They had significantly longer hospital stays (OR = 13.45, 95% CI = 2.86–63.22, *p* = 0.001 in the univariate analysis) and more frequent ICU admissions (OR = 1.88, 95% CI = 1.39–2.55, *p* < 0.001 in the univariate analysis). Longer hospital stays in liquid tumor patients may also be a result of their vulnerability to infection or inpatient administration of chemotherapeutics. Nevertheless, the increased frequency of ICU admissions undoubtedly reflects greater disease severity. We assumed this higher morbidity and mortality may largely be due to underlying thrombocytopenia; in our multivariable analysis of in-hospital mortality, every incremental drop in platelet count of 10 K/uL increased the mortality risk by 4%, and we know that patients with hematologic malignancies often have platelet counts below 50 K/uL, with some cases reaching below 20 K/uL. Indeed, our patients with platelet counts <50 K/uL had around three-fold risk of in-hospital mortality (OR = 3.41, 95% CI = 2.24–5.23, *p* < 0.001), 7-day mortality (OR = 2.57, 95% CI = 1.60–4.13, *p* < 0.001) and 30-day mortality (OR = 2.24, 95% CI = 1.57–3.19, *p* < 0.001). Patients with platelet counts <50 K/uL had also higher likelihood of ICU admission (OR = 2.22, 95% CI = 1.60–3.09, *p* < 0.001) and longer hospital length of stay (OR = 27.96, 95% CI = 5.20–150.4, *p* < 0.001).

Nearly half of our patients with ICH presented with headache, either alone or accompanied with other symptoms such as altered mental status. There is little debate that new neurologic deficits require imaging in the ED, but in the general population, judicious use of imaging to evaluate headache is recommended using clinical decision tools such as the Canadian computed tomography head rule or the Ottawa subarachnoid hemorrhage [15,16]. In the cancer population, such screening is not validated, and the frequency of ICH in patients presenting with non-focal symptoms such as headache or altered mental status makes bypassing imaging risky.

In fact, diagnostic imaging findings proved to be an important predictive variable for outcomes. Herniation and hydrocephalus were the most significant worrisome radiologic findings predicting short-term mortality, yet both were much less common than cerebral edema and/or midline shift. Intra-metastatic bleeding seems to confer a lower risk of mortality compared with other risks such as hematologic malignancy, active cancer or therapy, and dysrhythmia. Although associated edema was greater for bleeding within tumors, there was less midline shift and herniation (Table S5).

Management of ICH in our cancer patients reflected the etiology of their bleeds. Less invasive interventions such as platelet or dexamethasone administration were most frequent, and surgical interventions were uncommon (11.7%). Few of our patients (9) were on anticoagulants requiring reversal agents such as protamine or vitamin K. Similarly, use of hemostatic agents such as prothrombin complex concentrate (Kcentra), factor VIIa or tranexamic acid was not reported. The rationale for such conservative management is not always clear; it may reflect a palliative approach in patients with a poor prognosis from advanced cancer, or it may be that thrombocytopenia was the more common problem.

The analysis of risk factors for poor outcomes is perhaps the most significant contribution of this study. Hypertension, a well-established risk factor for ICH in the general population, was also present in nearly half of our cancer patients and was associated with longer hospital stays and higher ICU admission rates. In contrast, intratumor bleeding, unique to cancer patients, was associated with the shorter length of hospital stay in the univariate analysis and with reduced ICU admission rates.

Dysrhythmia and anticoagulant use were also predictive of poorer outcomes. The fatality of ICH in non-cancer patients receiving anticoagulation therapy has been shown to often exceed 30% (prior to the use of direct-acting oral anticoagulants (DOACs) and low-molecular-weight heparin (LMWH)) [17]. Cancer patients frequently receive anticoagulants owing to the increased incidence of venous thromboembolism (VTE) in malignancy [18], which is estimated to be as high as 12.6% [19]. Patients receiving anticoagulants have been shown to have increased morbidity and mortality due to secondary hematoma expansion [20,21,22]. Yet in one large series of ICH in cancer patients, anticoagulation therapy did not confer higher mortality, even though more patients (19% compared with 13.8%) were receiving anticoagulants in that series than in the current study [12]. Similarly, in a 2017 comparison of outcomes of ICH in patients with and without cancer, few were receiving anticoagulants (8.1% in patients with cancer, 6.9% in those without) [7].

Our patients were screened for all available anticoagulants (Table S11), yet only enoxaparin was used with any frequency (12.8% compared with 1.3% for warfarin), and no patients were receiving rivaroxaban or apixaban, reflecting the standard treatment for VTE during the period studied. One cannot conclude from these data whether the low association between anticoagulation therapy and poor outcomes is due to judicious use of anticoagulants in this vulnerable population or due to the inherently lower risk of bleeding of these anticoagulants.

Thrombocytopenia significantly raised the risk of in-hospital, 7-day, and 30-day mortality. For each 10-unit drop in platelet count, the risk of in-hospital mortality rose by 4%, the risk of 7-day mortality by 3%, and the risk of 30-day mortality by 10% (Figure 2). Many of our cancer patients had severe thrombocytopenia due to chemotherapy or the malignancy itself, as in leukemia. The risk of spontaneous hemorrhage in severe thrombocytopenia is well recognized, and this is the rationale behind prophylactic platelet transfusions. Our study showed that thrombocytopenia remains one of the major contributors to poor outcome as measured by 7, 30 day, in-hospital mortality, ICU admission, and hospital LOS, despite best practices in prophylaxis [23,24,25]. Anemia was also common in our patient population and increased mortality risk, presumably owing to decreased oxygen-carrying capacity to the threatened brain tissue. Low hemoglobin levels at the time of acute ischemic stroke are associated with larger infarcts and increased infarct growth [26,27].

In the decade since the last study of ICH in cancer patients, many new therapies have increased the lifespan of patients with liquid tumors, and new anticoagulant classes such as low-molecular-weight heparin have made anticoagulation therapy safer compared with vitamin K inhibitors. Concomitantly, targeted and immune-mediated therapies have decreased the incidence of treatment-related coagulopathies. Perhaps this has been protective against the risk and/or severity of ICH in cancer patients. Although 66.9% of our patients were on active therapy, and over 90% had either stage IV cancer or a hematologic malignancy, our in-hospital mortality was only 15.1% and 30-day mortality was 25.6%.

Our large sample size mitigated many study biases, but some remained. Foremost, this was a single-center retrospective study with a singular patient population and perhaps treatment preferences, although our demographics were very similar to those of previous studies [7,12]. We had only surrogate markers for severity of neurologic dysfunction upon presentation, and because this was a retrospective study, the Glasgow Coma Scale or Rankin scores for objectively measuring the clinical severity of the bleed were not consistently available, we could only report on mortality with confidence. Similarly, the presenting symptoms lacked specificity, for example, not distinguishing between focal paresis and generalized weakness. When more than 20% of the patients studied had missing values for laboratory biomarkers we did not included them in the analysis. Some of these, including the plasmatic coagulation international normalized ratio (INR), could be important as some cancer patients might have liver dysfunction or alimentary vitamin K deficiency. We used pharmacy data of anticoagulant use as a surrogate for coagulopathy, which did not account for all causes of coagulopathy that can be measured by prothrombin time, partial thromboplastin time, thrombin time and fibrinogen. Our objective data and radiographic, medication, and laboratory findings, however, generated solid multivariable analyses.

Although patients with intratumor bleeding appeared to have lower mortality rates, the ***p*** value did not reach significance, perhaps because the number of patients in this subgroup was inadequate. Additionally, some radiologic features of the intracranial hemorrhage, such as the hematoma size/volume or type of hydrocephalus, could not be described as several reports lacked such information. Further studies are needed to better describe these characteristics’ and determine their association with different ICH outcomes.

The study period was before the advent of DOACs for the treatment of VTE in cancer and atrial fibrillation, so we could not incorporate the presumed decreased risk of ICH. We were able to associate ICH risk factors with outcomes but not the prevalence of ICH because we did not have the denominator of all patients with and without ICH for particular risk factors. We do not have data on the frequency of repeat ED visits for specific malignancies or proportionally how many were being treated at MD Anderson. Finally, not all the cancer patients with ICH presented at our ED; some went to other hospitals, some bypassed the ED when admitted, and some, presumably the least symptomatic, may never have been diagnosed at all. Nevertheless, we believe the outcomes data offered here will help stratify the risk of morbidity and mortality in cancer patients presenting with ICH.

## 5. Conclusions

ICH remains an uncommon diagnosis among the ailments that bring cancer patients to the ED. In our cohort, in-hospital, 7-day, and 30-day mortality rates were lower than previously reported, and about two-thirds of patients were discharged to home, suggesting limited morbidity or disability from their hemorrhage. Among the multiple known risk factors and clinical characteristics of ICH, the ones most helpful in predicting patient outcomes were hypertension, low platelet count, and diagnostic imaging. Understanding how the clinical presentation, risk factors, and imaging findings correlate with patient morbidity and mortality is helpful in guiding diagnostic evaluation of ICH and the aggressiveness of care.

## Figures and Tables

**Table 1:** Demographic and clinical characteristics of cancer patients with ICH.

Characteristic	No. of Patients (%)			*p*
	Total	Spontaneous ICH	Traumatic ICH	
Total	704	576	128	
Median age (IQR), years	61 (49–70)	59 (48–68)	66 (58–74)	**<0.001**
Sex				0.624
Female	330 (46.9)	267 (46.4)	63 (49.2)	
Male	374 (53.1)	309 (53.6)	65 (50.8)	
Race				0.156
Non-White	204 (29.0)	174 (30.2)	30 (23.4)	
White	500 (71.0)	402 (69.8)	98 (76.6)	
Median CCI (IQR)	7 (4–9)	7 (4–9)	7 (4–9)	0.581
Cancer type				**<0.001**
Brain and spinal cord	89 (12.6)	77 (13.4)	12 (9.4)	
Hematologic	250 (35.5)	180 (31.3)	70 (54.7)	
Leukemia	196 (27.8)	143 (24.8)	53 (41.4)	
Lymphoma	30 (4.3)	25 (4.3)	5 (3.9)	
Multiple myeloma	24 (3.4)	12 (2.1)	12 (9.4)	
Solid tumors	365 (51.8)	319 (55.4)	46 (35.9)	
Melanoma	123 (17.5)	114 (19.8)	9 (7.0)	
Lung	65 (9.2)	59 (10.2)	6 (4.7)	
Breast	37 (5.3)	31 (5.4)	6 (4.7)	
Gastrointestinal	36 (5.1)	29 (5.0)	7 (5.5)	
Sarcoma	23 (3.3)	22 (3.8)	1 (0.8)	
Head and neck	21 (3.0)	12 (2.1)	9 (7.0)	
Renal	16 (2.3)	16 (2.8)	0 (0.0)	
Thyroid	8 (1.1)	8 (1.4)	0 (0.0)	
Other	36 (5.1)	28 (4.9)	8 (6.2)	
Cancer stage				**<0.001**
I	12 (1.7)	11 (1.9)	1 (0.8)	
II	20 (2.8)	14 (2.4)	6 (4.7)	
III	29 (4.1)	19 (3.3)	10 (7.8)	
IV	393 (55.8)	352 (61.1)	41 (32.0)	
Hematologic	250 (35.5)	180 (31.3)	70 (54.7)	
Cancer status				**0.012**
Stable	79 (11.2)	56 (9.7)	23 (18.0)	
Active	625 (88.8)	520 (90.3)	105 (82.0)	
Active cancer treatment				0.203
No	233 (33.1)	184 (31.9)	49 (38.3)	
Yes	471 (66.9)	392 (68.1)	79 (61.7)	
History of VTE				0.227
No	588 (83.5)	476 (82.6)	112 (87.5)	
Yes	116 (16.5)	100 (17.4)	16 (12.5)	
Hypertension				**0.034**
No	392 (55.7)	332 (57.6)	60 (46.9)	
Yes	312 (44.3)	244 (42.4)	68 (53.1)	
Hypercholesterolemia				**0.008**
No	568 (80.7)	476 (82.6)	92 (71.9)	
Yes	136 (19.3)	100 (17.4)	36 (28.1)	
Smoking				**0.034**
No	683 (97.0)	563 (97.7)	120 (93.8)	
Yes	21 (3.0)	13 (2.3)	8 (6.3)	
Dysrhythmia				0.060
No	643 (91.3)	532 (92.4)	111 (86.7)	
Yes	61 (8.7)	44 (7.6)	17 (13.3)	
Antiplatelets within 90 days ^†^				0.060
No	665 (94.5)	549 (95.3)	116 (90.6)	
Yes	39 (5.5)	27 (4.7)	12 (9.4)	
Anticoagulants within 90 days ^†^				1.000
No	607 (86.2)	497 (86.3)	110 (85.9)	
Yes	97 (13.8)	79 (13.7)	18 (14.1)	
Median hemoglobin (IQR), g/dL ^‡^	11.0 (9.3, 12.9)	11.3 (9.4, 13.1)	10.1 (8.8, 12.2)	**<0.001**
Median platelet count (IQR), K/uL ^‡^	150 (35, 235)	159 (42, 242)	75 (20, 193)	**<0.001**

Abbreviations: ICH, intracranial hemorrhage; IQR, interquartile range; CCI, Charlson comorbidity index; VTE, venous thromboembolism. ^†^ Based on pharmacy data within 90 days of presentation. ^‡^ Local normal values: Hemoglobin (12.0–16.0 gm/dL) and platelet count (140–440 K/uL).

**Table 2:** Management and outcome of ICH in cancer patients.

Characteristic	No. of Patients (%)			*p*
	Total	Spontaneous ICH	Traumatic ICH	
Management				
Initial treatment or intervention				0.313
Observation	44 (6.2)	33 (5.7)	11 (8.6)	
Active ^†^	660 (93.8)	543 (94.3)	117 (91.4)	
Platelets	211 (30.0)	161 (28.0)	50 (39.1)	
Dexamethasone	143 (20.3)	134 (23.3)	9 (7.0)	
Neurosurgery	124 (17.6)	109 (18.9)	15 (11.7)	
Discontinue anticoagulant	56 (8.0)	49 (8.5)	7 (5.5)	
Antihypertensive medications	60 (8.5)	48 (8.3)	12 (9.4)	
FFP	56 (8.0)	38 (6.6)	18 (14.1)	
Intubation/hyperventilation	37 (5.3)	33 (5.7)	4 (3.1)	
XRT	19 (2.7)	19 (3.3)	0 (0.0)	
Mannitol	17 (2.4)	14 (2.4)	3 (2.3)	
Protamine	8 (1.1)	8 (1.4)	0 (0.0)	
Hypertonic saline	6 (0.9)	6 (1.0)	0 (0.0)	
cryoprecipitate	3 (0.4)	2 (0.3)	1 (0.8)	
Vitamin K	1 (0.1)	0 (0.0)	1 (0.8)	
Hospital-related outcomes				
Final disposition from the ED				0.455
Floor	356 (50.6)	300 (52.1)	56 (43.8)	
ICU	292 (41.5)	230 (39.9)	62 (48.4)	
Home	33 (4.7)	27 (4.7)	6 (4.7)	
Operation room	6 (0.9)	4 (0.7)	2 (1.6)	
Telemetry or CDU	5 (0.7)	4 (0.7)	1 (0.8)	
Dead	4 (0.6)	4 (0.7)	0 (0.0)	
Palliative care unit	4 (0.6)	4 (0.7)	0 (0.0)	
Hospice	2 (0.3)	1 (0.2)	1 (0.8)	
Transfer	2 (0.3)	2 (0.3)	0 (0.0)	
Final disposition from the hospital				**0.008**
Home	437 (62.1)	369 (64.1)	68 (53.1)	
Dead	106 (15.1)	80 (13.9)	26 (20.3)	
Hospice	81 (11.5)	70 (12.2)	11 (8.6)	
Discharged from the ED	37 (5.3)	30 (5.2)	7 (5.5)	
Nursing facility	17 (2.4)	9 (1.6)	8 (6.3)	
Transfer	11 (1.6)	7 (1.2)	4 (3.1)	
Rehabilitation facility	10 (1.4)	7 (1.2)	3 (2.3)	
Long-term acute care facility	5 (0.7)	4 (0.7)	1 (0.8)	
ED length of stay (IQR), hours	8 (5–10)	8 (5–10)	8 (6–11)	0.407
ICU admission				0.095
No	412 (58.5)	346 (60.1)	66 (51.6)	
Yes	292 (41.5)	230 (39.9)	62 (48.4)	
Median hospital length of stay, days (IQR) ^‡^	5(3–11)	5 (3–10)	6 (4–13)	**0.007**
Median ICU length of stay (IQR, days) ^§^	2 (1–4)	2 (1–3)	3 (2–5)	0.154
Mortality				
In-hospital				0.089
No	598 (84.9)	496 (86.1)	102 (79.7)	
Yes	106 (15.1)	80 (13.9)	26 (20.3)	
7-days				0.743
No	624 (88.6)	509 (88.4)	115 (89.8)	
Yes	80 (11.4)	67 (11.6)	13 (10.2)	
30-days				0.404
No	524 (74.4)	433 (75.1)	91 (71.1)	
Yes	180 (25.6)	143 (24.8)	37 (28.9)	

Abbreviations: ICH, intracranial hemorrhage; FFP, fresh frozen plasma; XRT, radiotherapy; ED, emergency department; ICU, intensive care unit; CDU, clinical decision unit; IQR, interquartile range. ^†^ Numbers in active initial treatment/intervention do not add up to 100% because many patients had more than one initial treatment/intervention in the ED. XRT and neurosurgery were also considered initial treatment if they were requested by the related specialist during consultation in the ED. ^‡^ Only for patients who were admitted to the hospital (*n* = 663). ^§^ Only for patients admitted to the ICU (*n* = 292).

**Table 3:** Multivariable analysis of the association between clinical factors and hospital outcomes in cancer patients with intracranial hemorrhage.

Variable	Hospital LOS ^†^		ICU Admission	
	OR (95% CI)	*p*	OR (95% CI)	*p*
CCI	0.86 (0.63–1.19)	0.366	-	-
Hematologic malignancy (compared with solid tumors)	3.08 (0.38–24.70)	0.289	1.39 (0.96–2.01)	0.082
Hypertension	4.77 (1.30–22.70)	**0.045**	1.52 (1.09–2.12)	**0.013**
Hypercholesterolemia	-	-	1.22 (0.81–1.84)	0.346
Hemoglobin level, gm/dL	1.01 (0.70–1.45)	0.960	0.97 (0.90–1.05)	0.484
Intratumor bleeding	0.45 (0.08–2.71)	0.385	0.66 (0.45–0.97)	**0.033**
Platelet count (increment of 10 K/uL)	0.93 (0.87–1.00)	**0.041**	0.99 (0.97–1.01)	0.197

Abbreviations: LOS, length of stay; ICU, intensive care unit; OR, odds ratio; CI, confidence interval; CCI, Charlson comorbidity index. ^†^ Patients who died in the hospital were excluded.

**Table 4:** Multivariable analysis of the association between radiologic findings and short-term mortality in cancer patients with intracranial hemorrhage.

Radiologic Finding	In-Hospital Mortality		7-Day Mortality		30-Day Mortality	
	AOR ^†^ (95% CI)	*p*	AOR ^†^ (95% CI)	*p*	AOR ^†^ (95% CI)	*p*
Edema	2.27 (1.41–3.70)	**<0.001**	2.73 (1.61–4.67)	**<0.001**	1.78 (1.20–2.64)	**0.004**
Midline shift	2.59 (1.68–4.00)	**<0.001**	3.53 (2.18–5.81)	**<0.001**	1.85 (1.29–2.65)	**<0.001**
Herniation	7.76 (4.58–13.27)	**<0.001**	10.63 (6.18–18.48)	**<0.001**	5.83 (3.62–9.50)	**<0.001**
Hydrocephalus	3.83 (2.12–6.86)	**<0.001**	5.78 (3.19–10.39)	**<0.001**	3.71 (2.22–6.22)	**<0.001**

Abbreviations: AOR, adjusted odds ratio. ^†^ Adjusted for platelet count, hemoglobin level, and intratumor bleeding.

## Data Availability

The data presented in this study are available on request from the corresponding author. The data are not publicly available due and an IRB approval from MD Anderson Cancer Center is required to share the data.

## Supplementary Materials

The following supporting information can be downloaded at: https://www.mdpi.com/article/10.3390/jcm11030643/s1, Table S1: Presenting symptoms for cancer patients with intracranial hemorrhage (*n* = 704); Table S2: Characteristics of ICH in cancer patients; Table S3. Types of brain herniation in cancer patients presenting with intracranial hemorrhage; Table S4: Primary cancer type for the metastatic brain lesions; Table S5: Radiological findings stratified by bleeding type; S6: Univariate analysis of the association between clinical factors and hospital outcomes in cancer patients with intracranial hemorrhage; Table S7: Mortality in cancer patients with intracranial hemorrhage, stratified by cancer type; Table S9: Univariate analysis of the association between clinical factors and short-term mortality in cancer patients with intracranial hemorrhage; Table S10: Univariate analysis of the association between radiologic findings and short-term mortality in cancer patients with intracranial hemorrhage; Table S11: Anticoagulant agents (excluding heparin) used in cancer patients with ICH.

## References

[1] Krishnamurthi R.V., Moran A.E., Forouzanfar M.H., Bennett D.A., Mensah G.A., Lawes C.M., Barker-Collo S., Connor M., Roth G.A., Sacco R. (2014). The global burden of hemorrhagic stroke: A summary of findings from the gbd 2010 study. Glob. Heart.

[2] An S.J., Kim T.J., Yoon B.W. (2017). Epidemiology, risk factors, and clinical features of intracerebral hemorrhage: An update. J. Stroke.

[3] Garg R., Biller J. (2019). Recent advances in spontaneous intracerebral hemorrhage. F1000Research.

[4] Chu K.H., Mahmoud I., Hou X.Y., Winter C.D., Jeffree R.L., Brown N.J., Brown A.F. (2018). Incidence and outcome of subarachnoid haemorrhage in the general and emergency department populations in queensland from 2010 to 2014. Emerg. Med. Australas. EMA.

[5] González-Pérez A., Gaist D., Wallander M.A., McFeat G., García-Rodríguez L.A. (2013). Mortality after hemorrhagic stroke: Data from general practice (the health improvement network). Neurology.

[6] van Asch C.J., Luitse M.J., Rinkel G.J., van der Tweel I., Algra A., Klijn C.J. (2010). Incidence, case fatality, and functional outcome of intracerebral haemorrhage over time, according to age, sex, and ethnic origin: A systematic review and meta-analysis. Lancet Neurol..

[7] Murthy S.B., Shastri A., Merkler A.E., Hanley D.F., Ziai W.C., Fink M.E., Iadecola C., Kamel H., Navi B.B. (2016). Intracerebral hemorrhage outcomes in patients with systemic cancer. J. Stroke Cerebrovasc. Dis..

[8] Dayyani F., Mougalian S.S., Naqvi K., Shan J., Ravandi F., Cortes J., Weinberg J., Jabbour E., Faderl S., Wierda W. (2011). Prediction model for mortality after intracranial hemorrhage in patients with leukemia. Am. J. Hematol..

[9] Carney B.J., Uhlmann E.J., Puligandla M., Mantia C., Weber G.M., Neuberg D.S., Zwicker J.I. (2019). Intracranial hemorrhage with direct oral anticoagulants in patients with brain tumors. J. Thromb. Haemost. JTH.

[10] Zwicker J.I., Karp Leaf R., Carrier M. (2016). A meta-analysis of intracranial hemorrhage in patients with brain tumors receiving therapeutic anticoagulation. J. Thromb. Haemost. JTH.

[11] Donato J., Campigotto F., Uhlmann E.J., Coletti E., Neuberg D., Weber G.M., Zwicker J.I. (2015). Intracranial hemorrhage in patients with brain metastases treated with therapeutic enoxaparin: A matched cohort study. Blood.

[12] Navi B.B., Reichman J.S., Berlin D., Reiner A.S., Panageas K.S., Segal A.Z., DeAngelis L.M. (2010). Intracerebral and subarachnoid hemorrhage in patients with cancer. Neurology.

[13] Muster V., Gary T. (2021). Contrasts in glioblastoma-venous thromboembolism versus bleeding risk. Cells.

[14] Burth S., Ohmann M., Kronsteiner D., Kieser M., Löw S., Riedemann L., Laible M., Berberich A., Drüschler K., Rizos T. (2021). Prophylactic anticoagulation in patients with glioblastoma or brain metastases and atrial fibrillation: An increased risk for intracranial hemorrhage?. J. Neurooncol..

[15] Stiell I.G., Wells G.A., Vandemheen K., Clement C., Lesiuk H., Laupacis A., McKnight R.D., Verbeek R., Brison R., Cass D. (2001). The canadian ct head rule for patients with minor head injury. Lancet.

[16] Wu W.T., Pan H.Y., Wu K.H., Huang Y.S., Wu C.H., Cheng F.J. (2020). The ottawa subarachnoid hemorrhage clinical decision rule for classifying emergency department headache patients. Am. J. Emerg. Med..

[17] Qureshi A.I., Mendelow A.D., Hanley D.F. (2009). Intracerebral haemorrhage. Lancet.

[18] Qdaisat A., Wu W., Lin J.Z., Al Soud R., Yang Z., Hu Z., Gao S., Wu C.C., Liu X., Silvestre J. (2020). Clinical and cancer-related predictors for venous thromboembolism in cancer patients presenting to the emergency department. J. Emerg. Med..

[19] Khorana A.A., Dalal M., Lin J., Connolly G.C. (2013). Incidence and predictors of venous thromboembolism (vte) among ambulatory high-risk cancer patients undergoing chemotherapy in the united states. Cancer.

[20] Frontera J.A., Lewin J.J., Rabinstein A.A., Aisiku I.P., Alexandrov A.W., Cook A.M., del Zoppo G.J., Kumar M.A., Peerschke E.I., Stiefel M.F. (2016). Guideline for reversal of antithrombotics in intracranial hemorrhage: A statement for healthcare professionals from the neurocritical care society and society of critical care medicine. Neurocrit. Care.

[21] Flibotte J.J., Hagan N., O’Donnell J., Greenberg S.M., Rosand J. (2004). Warfarin, hematoma expansion, and outcome of intracerebral hemorrhage. Neurology.

[22] Adachi T., Hoshino H., Takagi M., Fujioka S. (2017). Volume and characteristics of intracerebral hemorrhage with direct oral anticoagulants in comparison with warfarin. Cerebrovasc. Dis. Extra.

[23] Chern J.J., Tsung A.J., Humphries W., Sawaya R., Lang F.F. (2011). Clinical outcome of leukemia patients with intracranial hemorrhage. Clinical article. J. Neurosurg..

[24] Gaydos L., Freireich E., Mantel N. (1962). The quantitative relation between platelet count and hemorrhage in patients with acute leukemia. N. Engl. J. Med..

[25] Mayda-Domac F., Misirli H., Yilmaz M. (2010). Prognostic role of mean platelet volume and platelet count in ischemic and hemorrhagic stroke. J. Stroke Cerebrovasc. Dis. Off. J. Natl. Stroke Assoc..

[26] Kimberly W.T., Wu O., Arsava E.M., Garg P., Ji R., Vangel M., Singhal A.B., Ay H., Sorensen A.G. (2011). Lower hemoglobin correlates with larger stroke volumes in acute ischemic stroke. Cerebrovasc. Dis. Extra.

[27] Tanne D., Molshatzki N., Merzeliak O., Tsabari R., Toashi M., Schwammenthal Y. (2010). Anemia status, hemoglobin concentration and outcome after acute stroke: A cohort study. BMC Neurol..

