# Supplementary material for: Characteristics and Outcomes of Intracranial Hemorrhage in Cancer Patients Visiting the Emergency Department
Source: J Clin Med. 2022 Jan 27;11(3):643. doi: 10.3390/jcm11030643 (PMC8837162; doi:10.3390/jcm11030643)
Supplement: Supplementary file 1 [file jcm-11-00643-s001.zip › jcm-1531031-supplementary.pdf]

**SUPPLEMENTAL MATERIAL**

**Table S1. Presenting symptoms for cancer patients with intracranial hemorrhage (n = 704)**

| Symptom | No. of patients (%) |
|---|---|
| Headache | 297 (42.2) |
| Altered mental status | 253 (35.9) |
| Paresis/weakness | 185 (26.3) |
| Nausea/vomiting | 145 (20.6) |
| Fall/trauma | 122 (17.3) |
| Speech changes | 79 (11.2) |
| Seizure | 72 (10.2) |
| Dizziness | 64 (9.1) |
| Visual changes | 45 (6.4) |
| Loss of consciousness | 31 (4.4) |
| Numbness | 20 (2.8) |

**Table S2. Characteristics of ICH in cancer patients**

| | No. of patients (%) | | | |
|---|---|---|---|---|
| **Characteristic** | **Total** | **Spontaneous ICH** | **Traumatic ICH** | ***P*** |
| Total | 704 | 576 | 128 | |
| ICH location | | | | **<0.001** |
| Subdural hematoma | 248 (35.2) | 169 (29.3) | 79 (61.7) | |
| Hemorrhagic metastasis | 199 (28.3) | 192 (33.3) | 7 (5.5) | |
| Intraparenchymal hemorrhage | 74 (10.5) | 68 (11.8) | 6 (4.7) | |
| Subarachnoid hemorrhage | 62 (8.8) | 41 (7.1) | 21 (16.4) | |
| Hemorrhage in primary tumor | 40 (5.7) | 36 (6.3) | 4 (3.1) | |
| Intraventricular hemorrhage | 25 (3.6) | 19 (3.3) | 6 (4.7) | |
| Postsurgical hemorrhage | 17 (2.4) | 17 (3.0) | 0 (0.0) | |
| Cerebellar hemorrhage | 17 (2.4) | 16 (2.8) | 1 (0.8) | |
| Basal ganglia hemorrhage | 9 (1.3) | 8 (1.4) | 1 (0.8) | |
| Brainstem hemorrhage | 3 (0.4) | 3 (0.5) | 0 (0.0) | |
| Epidural hematoma | 8 (1.1) | 5 (0.9) | 3 (2.3) | |
| Thalamic hemorrhage | 2 (0.3) | 2 (0.3) | 0 (0.0) | |
| Associated edema | | | | **<0.001** |
| No | 372 (52.8) | 277 (48.1) | 95 (74.2) | |
| Yes | 332 (47.2) | 299 (51.9) | 33 (25.8) | |
| Midline shift | | | | 0.087 |
| No | 463 (65.8) | 370 (64.2) | 93 (72.7) | |
| Yes | 241 (34.2) | 206 (35.8) | 35 (27.3) | |
| Herniation | | | | 0.646 |
| No | 611 (86.8) | 502 (87.2) | 109 (85.2) | |
| Yes | 93 (13.2) | 74 (12.8) | 19 (14.8) | |
| Hydrocephalus | | | | 0.434 |
| No | 627 (89.1) | 510 (88.5) | 117 (91.4) | |
| Yes | 77 (10.9) | 66 (11.5) | 11 (8.6) | |

Abbreviations: ICH, intracranial hemorrhage. Boldface indicates statistical significance.

**Table S3. Types of brain herniation in cancer patients presenting with intracranial hemorrhage**

| Herniation type | No. of patients* |
|---|---|
| Subfalcine herniation | 35 (5.0%) |
| Uncal herniation | 30 (4.3%) |
| Transtentorial except Uncal herniation | 19 (2.7%) |
| Cerebellar tonsillar herniation | 14 (2.0%) |
| Others or undefined | 17 (2.4%) |

*Some patients had more than one type of herniation at the same time

**Table S4. Primary cancer type for the metastatic brain lesions**

| Primary cancer type | No. of patients (%) |
|---|---|
| Melanoma | 94 (47.2) |
| Lung | 37 (18.6) |
| Breast | 15 (7.5) |
| Sarcoma | 12 (6.0) |
| Kidney | 7 (3.5) |
| Thyroid | 6 (3.0) |
| Gastrointestinal | 6 (3.0) |
| Head and neck | 4 (2.0) |
| Others | 18 (9.0) |

**Table S5. Radiological findings stratified by bleeding type**

| Radiological finding | No. of patients (%) | | | *P* |
|---|---|---|---|---|
| | Intra-metastatic bleeding | Intra-primary tumor bleeding | Other bleeds | |
| Associated edema | | | | <0.001 |
| No | 48 (24.1) | 13 (32.5) | 311 (66.9) | |
| Yes | 151 (75.9) | 27 (67.5) | 154 (33.1) | |
| Midline shift | | | | 0.006 |
| No | 146 (73.4) | 20 (50.0) | 297 (63.9) | |
| Yes | 53 (26.6) | 20 (50.0) | 168 (36.1) | |
| Herniation | | | | 0.228 |
| No | 178 (89.4) | 32 (80.0) | 401 (86.2) | |
| Yes | 21 (10.6) | 8 (20.0) | 64 (13.8) | |
| Hydrocephalus | | | | 0.462 |
| No | 173 (86.9) | 35 (87.5) | 419 (90.1) | |
| Yes | 26 (13.1) | 5 (12.5) | 46 (9.9) | |

**Table S6. Univariate analysis of the association between clinical factors and hospital outcomes in cancer patients with intracranial hemorrhage**

| Variable | Hospital LOS† | | ICU admission | |
|---|---|---|---|---|
| | OR (95% CI) | *P* | OR (95% CI) | *P* |
| Age <65 years (compared with ≥65 years) | 0.71 (0.14-3.53) | 0.677 | 0.98 (0.72-1.34) | 0.904 |
| Male (compared with female) | 0.86 (0.18-4.07) | 0.846 | 0.81 (0.60-1.09) | 0.162 |
| White (compared with non-White) | 0.82 (0.15-4.53) | 0.818 | 0.96 (0.69-1.34) | 0.815 |
| CCI | 0.66 (0.50-0.86) | **0.002** | 0.98 (0.93-1.03) | 0.372 |
| Hematologic malignancy (compared with solid tumors) | 13.45 (2.86-63.22) | **0.001** | 1.88 (1.39-2.55) | **<0.001** |
| Active cancer status | 0.16 (0.01-1.96) | 0.150 | 0.71 (0.43-1.14) | 0.164 |
| Active cancer therapy | 0.34 (0.07-1.78) | 0.202 | 0.89 (0.65-1.23) | 0.479 |
| Hypertension | 4.02 (0.85-19.09) | 0.079 | 1.63 (1.20-2.21) | **0.002** |
| Hypercholesterolemia | 1.08 (0.16-7.45) | 0.939 | 1.48 (1.02-2.16) | 0.041 |
| Current smoker | 0.1 (0.00-11.85) | 0.340 | 1.29 (0.53-3.11) | 0.563 |
| Dysrhythmia | 9.94 (0.60-163.39) | 0.108 | 1.22 (0.71-2.06) | 0.464 |
| Antiplatelets used within 90 days | 1.31 (0.04-41.39) | 0.879 | 1.22 (0.63-2.34) | 0.542 |
| Anticoagulants used within 90 days | 0.71 (0.08-6.66) | 0.766 | 0.69 (0.44-1.08) | 0.110 |
| Hemoglobin level, gm/dL | 0.73 (0.53-1.01) | 0.057 | 0.90 (0.85-0.96) | **0.002** |
| Intratumor bleeding | 0.10 (0.02-0.49) | **0.005** | 0.47 (0.33-0.65) | **<0.001** |
| Platelet count (increment of 10) | 0.89 (0.84-0.95) | **<0.001** | 0.98 (0.96-0.99) | **<0.001** |
| Platelet count <50 (compared with ≥50) | 27.96 (5.20-150.4) | **<0.001** | 2.22 (1.60-3.09) | **<0.001** |

*Abbreviations: LOS, length of stay; ICU, intensive care unit; OR, odds ratio; CI, confidence interval; CCI, Charlson comorbidity index.

†Patients who died in the hospital were excluded.

**Table S7. Mortality in cancer patients with intracranial hemorrhage, stratified by cancer type**

| Cancer type | No. of patients | No. of deaths (% of subgroup) | | |
|---|---|---|---|---|
| | | 7-day mortality | 30-day mortality | 1-year mortality |
| Brain and spinal cord | 89 | 6 (6.7) | 13 (14.6) | 49 (55.1) |
| Breast | 37 | 2 (5.4) | 7 (18.9) | 19 (51.4) |
| Gastrointestinal | 36 | 6 (16.7) | 10 (27.8) | 29 (80.6) |
| Head and neck | 21 | 1 (4.8) | 6 (28.6) | 13 (61.9) |
| Leukemia | 196 | 31 (15.8) | 63 (32.1) | 138 (70.4) |
| Lung | 65 | 7 (10.8) | 17 (26.2) | 56 (86.2) |
| Lymphoma | 30 | 1 (3.3) | 4 (13.3) | 15 (50.0) |
| Melanoma | 123 | 13 (10.6) | 29 (23.6) | 91 (74.0) |
| Multiple myeloma | 24 | 6 (25.0) | 12 (50.0) | 17 (70.8) |
| Other | 60 | 3 (5.0) | 9 (15.0) | 31 (51.7) |
| Sarcoma | 23 | 4 (17.4) | 10 (43.5) | 18 (78.3) |

**Table S8. Mortality rates and ICU admission stratified by intracranial hemorrhage type**

| **Outcome** | **Intraparenchymal hemorrhage** | **Subdural hematoma** | **Intraventricular hemorrhage** | **Hemorrhagic tumor** | **Subarachnoid hemorrhage** | **Others** | ***P*** |
|---|---|---|---|---|---|---|---|
| Total | 74 | 248 | 25 | 239 | 62 | 56 | |
| In-hospital mortality | 25 (33.8) | 45 (18.1) | 2 (8.0) | 18 (7.5) | 8 (12.9) | 8 (14.3) | <0.001 |
| 7-days mortality | 21 (28.4) | 26 (10.5) | 3 (12.0) | 17 (7.1) | 6 (9.7) | 7 (12.5) | <0.001 |
| 30-days mortality | 31 (41.9) | 65 (26.2) | 7 (28.0) | 57 (23.9) | 10 (16.1) | 10 (17.9) | 0.009 |
| ICU admission | 44 (59.5) | 107 (43.1) | 15 (60.0) | 71 (29.7) | 31 (50.0) | 24 (42.9) | <0.001 |

*Abbreviations: ICU, intensive care unit.

**Table S9. Univariate analysis of the association between clinical factors and short-term mortality in cancer patients with intracranial hemorrhage**

| | In-hospital mortality | | 7-day mortality | | 30-day mortality | |
|---|---|---|---|---|---|---|
| **Variable** | **OR (95% CI)** | ***P*** | **OR (95% CI)** | ***P*** | **OR (95% CI)** | ***P*** |
| Age <65 years (compared with ≥65 years) | 0.80 (0.53-1.22) | 0.290 | 0.67 (0.42-1.07) | 0.093 | 0.85 (0.60-1.21) | 0.366 |
| Male (compared with female) | 0.79 (0.52-1.19) | 0.263 | 0.69 (0.43-1.11) | 0.126 | 0.73 (0.52-1.02) | 0.069 |
| White (compared with non-White) | 0.89 (0.57-1.40) | 0.596 | 1.17 (0.70-2.02) | 0.562 | 1.05 (0.72-1.53) | 0.814 |
| CCI | 0.98 (0.92-1.06) | 0.676 | 0.99 (0.92-1.08) | 0.884 | 1.04 (0.98-1.10) | 0.178 |
| Hematologic malignancy (compared with solid tumors) | 2.17 (1.42-3.36) | **<0.001** | 1.37 (0.86-2.19) | 0.189 | 1.18 (0.84-1.65) | 0.346 |
| Active cancer status | 0.90 (0.44-1.70) | 0.765 | 0.73 (0.30-1.55) | 0.456 | 0.54 (0.28-0.97) | 0.051 |
| Active cancer therapy | 1.23 (0.79-1.96) | 0.361 | 1.26 (0.76-2.14) | 0.376 | 1.62 (1.12-2.39) | **0.013** |
| Hypertension | 1.05 (0.69-1.58) | 0.828 | 1.10 (0.68-1.75) | 0.701 | 0.98 (0.70-1.38) | 0.913 |
| Current smoker | 1.34 (0.38-3.71) | 0.605 | 1.31 (0.30-3.98) | 0.671 | 0.91 (0.29-2.35) | 0.848 |
| Dysrhythmia | 1.43 (0.70-2.71) | 0.294 | 1.83 (0.87-3.56) | 0.091 | 1.14 (0.62-2.01) | 0.672 |
| Antiplatelets used within 90 days | 1.49 (0.62-3.20) | 0.330 | 0.64 (0.15-1.82) | 0.459 | 0.74 (0.31-1.56) | 0.455 |
| Anticoagulants used within 90 days | 1.04 (0.55-1.83) | 0.904 | 0.88 (0.41-1.70) | 0.721 | 0.95 (0.57-1.54) | 0.834 |
| Intratumor bleeding | 0.35 (0.20-0.58) | **<0.001** | 0.49 (0.27-0.84) | **0.013** | 0.88 (0.61-1.25) | 0.472 |
| Platelet count (increment of 10) | 0.95 (0.93-0.97) | **<0.001** | 0.97 (0.95-0.99) | **0.003** | 0.97 (0.96-0.99) | **<0.001** |
| Platelet count <50 (compared with ≥50) | 3.41 (2.24-5.23) | **<0.001** | 2.57 (1.60-4.13) | **<0.001** | 2.24 (1.57-3.19) | **<0.001** |
| Hemoglobin level, gm/dL | 0.83 (0.76-0.91) | **<0.001** | 0.90 (0.81-0.99) | **0.036** | 0.86 (0.80-0.93) | **<0.001** |

*Abbreviations: OR, odds ratio; CI, confidence interval; CCI, Charlson comorbidity index. Boldface indicates statistical significance.

**Table S10. Univariate analysis of the association between radiologic findings and short-term mortality in cancer patients with intracranial hemorrhage**

| | In-hospital mortality | | 7-day mortality | | 30-day mortality | |
|---|---|---|---|---|---|---|
| **Radiologic finding** | **OR (95% CI)** | ***P**** | **OR (95% CI)** | ***P**** | **OR (95% CI)** | ***P**** |
| Edema | 1.14 (0.76-1.73) | 0.525 | 1.6 (1.01-2.58) | **0.049** | 1.28 (0.91-1.80) | 0.154 |
| Midline shift | 2.39 (1.57-3.64) | **<0.001** | 3.37 (2.10-5.48) | **<0.001** | 1.65 (1.16-2.34) | **0.005** |
| Herniation | 6.23 (3.84-10.11) | **<0.001** | 9.34 (5.56-15.75) | **<0.001** | 4.78 (3.04-7.55) | **<0.001** |
| Hydrocephalus | 2.79 (1.60-4.74) | **<0.001** | 4.61 (2.62-7.98) | **<0.001** | 2.94 (1.80-4.77) | **<0.001** |

*Boldface indicates statistical significance

**Table S11. Anticoagulant agents (excluding heparin) used in cancer patients with ICH**

| | No. of patients (%) | | |
|---|---|---|---|
| **Anticoagulant agent** | **Total, n=704** | **Spontaneous ICH, n=576** | **Traumatic ICH, n=128** |
| Enoxaparin | 90 (12.8) | 73 (12.7) | 17 (13.3) |
| Dalteparin | 5 (0.7) | 5 (0.9) | 0 (0.0) |
| Warfarin | 9 (1.3) | 3 (0.5) | 6 (4.7) |

*Abbreviations: ICH, intracranial hemorrhage.
